# Involvement of cerebellum in frontotemporal dementia: A case presentation using fluorodeoxyglucose positron emission tomography/magnetic resonance imaging (FDG-PET/MRI)

**Published:** 2019-01-05

**Authors:** Samson Nivins, Samuel Berkins

**Affiliations:** 1 Department of Molecular Imaging, Nueclear Healthcare Limited, Mumbai, India; 2 Department of Biomedical Engineering, Vellore Institute of Technology University, Chennai, India

**Keywords:** Dementia, Positron Emission Tomography, Magnetic Resonance Imaging

Frontotemporal dementia (FTD) is a neurodegenerative disorder marked by the focal degeneration of frontal and anterior temporal lobes.^1^ It is the second most common dementia affecting individuals under 65 years of age,^2^ and third most common form of dementia for individuals over 65 years of age.^3^ FTD is associated with early behavioural abnormalities, including apathy, disinhibition, obsessive and compulsive behaviours, emotional blunting, and loss of sympathy and empathy. Cognitive functions such as memory are relatively preserved in the early stages of the disease.^4^ The current diagnosis is based on the complete clinical presentations, while the subtypes tend to merge as the disease progress. 

Previous magnetic resonance imaging (MRI) studies on patients with FTD showed atrophy in the frontotemporal cortex with relative sparing of posterior cortical regions.^5^ In addition, fluorodeoxyglucose positron emission tomography (FDG-PET) documented decreased metabolism in the frontal, anterior temporal cortex, and subcortical structures.^6^ In this report, we present a 60-year-old man clinically diagnosed with FTD. The patient underwent simultaneous FDG-PET/MRI (Figure 1). 

In the present case, reduced glucose metabolism was observed in bilateral frontal and anterior cingulate cortex, as well as left temporal and parietotemporal cortex. 

**Figure 1:**
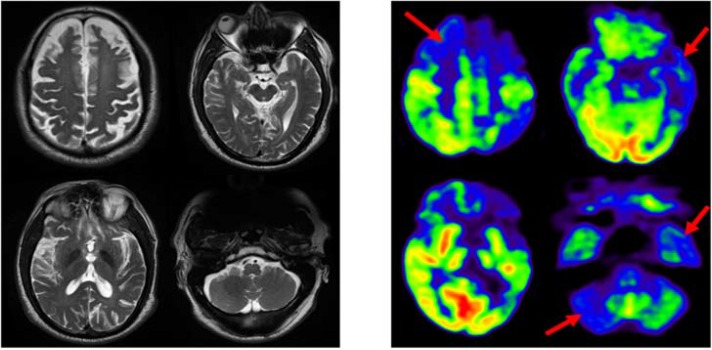
Fluorodeoxyglucose positron emission tomography/magnetic resonance imaging (FDG-PET/MRI) images of a 60-year-old man clinically diagnosed with frontotemporal dementia (FTD). MRI images are presented on the left side showing atrophy of the frontal, parietal, and temporal cortex. PET images are demonstrated on the right side showing decreased glucose metabolism in the bilateral frontal cortex, anterior cingulate cortex, left parietotemporal cortex, lateral temporal cortex, superior temporal cortex, medial temporal cortex, and right cerebellum.

This reduced metabolism in patients with FTD might be associated with the neuronal loss and astrocytosis in the frontal and temporal cortex as observed in previous pathological studies.^7^ In addition, this also could be related with the proteinopathy characterized by the presence of abnormal ubiquitinated protein inclusion in cytoplasm or nuclei of neuronal and glial cells.^8^ Posterior cingulate cortex was well preserved. The above-mentioned regions were previously documented in earlier literatures.^9^^-^^11^ Apart from the previously established pattern of reduced metabolism in FTD, cerebellum also showed reduced metabolism in the right side of the brain (pattern of crossed cerebellar diaschisis). Previous studies on FTD using FDG-PET has observed no involvement of cerebellum, and also considered cerebellum as a reference region for quantification.^9^^,^^12^ Furthermore, asymmetry was also noted in both cortical and subcortical regions.

Thus the present case brings to the notice that cerebellum pathology also need to be considered in patients with FTD. However, larger cohort study is needed for better understanding of the cerebellum involvement.

## Notes:

**How to cite this article: **Nivins S, Berkins S. Involvement of cerebellum in frontotemporal dementia: A case presentation using fluorodeoxyglucose positron emission tomography/magnetic resonance imaging (FDG-PET/MRI). Iran J Neurol 2019; 18(1): 38-40.
